# Paget’s disease of bone: pathogenesis, ocular manifestations, and therapeutic strategies

**DOI:** 10.1186/s13023-026-04412-4

**Published:** 2026-05-30

**Authors:** Bei Li, Yifan Kang

**Affiliations:** 1 Department of Ophthalmology, Chengdu University of Traditional Chinese Medicine Affiliated Integrated TCM&Western Medicine Hospital, Chengdu First People’s Hospital, Chengdu Integrated TCM & Western Medicine Hospital, No.18 Wanxiang North Road, Chengdu, Sichuan Province 610041 China; 2 Eye School of Chengdu, University of Traditional Chinese Medicine, Chengdu, Sichuan Province China

**Keywords:** Paget's disease of bone, Pathogenesis, Ocular manifestations, Treatment, Multidisciplinary management

## Abstract

**Objective:**

Paget’s disease of bone (PDB) is a chronic, focal metabolic bone disorder characterized by increased bone destruction and disordered bone formation, and is a rare disease in Asian populations. This review aims to systematically summarize the pathogenesis, ocular manifestations, and treatment strategies for PDB, with the goal of enhancing the understanding of its multi-system involvement and exploring more effective clinical management approaches.

**Methods:**

A systematic search of electronic databases, including PubMed, was conducted using Boolean operators to combine keywords such as “Paget’s disease of bone”, “osteitis deformans”, “pathogenesis”, “disease mechanism”, “ocular”, “oculus”, “ophthalmic”, “eye”, “treatment”, “pharmacotherapy”, “medication”, etc. Following an initial screening and the exclusion of duplicate records, relevant articles were selected for comprehensive analysis. The level of evidence was assessed.

**Results:**

68 articles were included. This review elaborates on the roles of genetic and environmental factors in PDB and details its diverse ocular manifestations, including ptosis, cataract, macular disease, angioid streaks, optic neuropathy, and orbital disease. It also summarizes advances in current pharmacotherapy, primarily based on bisphosphonates, and other potential treatment strategies.

**Conclusion:**

Abnormal bone remodeling, driven by a combination of genetic susceptibility and environmental factors, constitutes the common pathological basis for both systemic skeletal lesions and specific ocular complications in PDB. Management should be based on multidisciplinary collaboration, integrating pharmacological intervention with individualized ocular care to control disease progression and prevent visual impairment.

## Highlights

This review provides the first systematic synthesis of the full spectrum of ocular manifestations in Paget’s disease of bone (PDB), from ptosis to vision-threatening complications.

It integrates current genetic and molecular mechanisms of PDB with their clinical implications for eye involvement and future targeted therapies.

A novel, risk-stratified management algorithm is proposed to guide screening, monitoring, and intervention for ocular PDB.

The review emphasizes a paradigm shift toward preventive, multidisciplinary care to preserve vision and improve quality of life in PDB patients.

## Search method

**Fig. 1:**
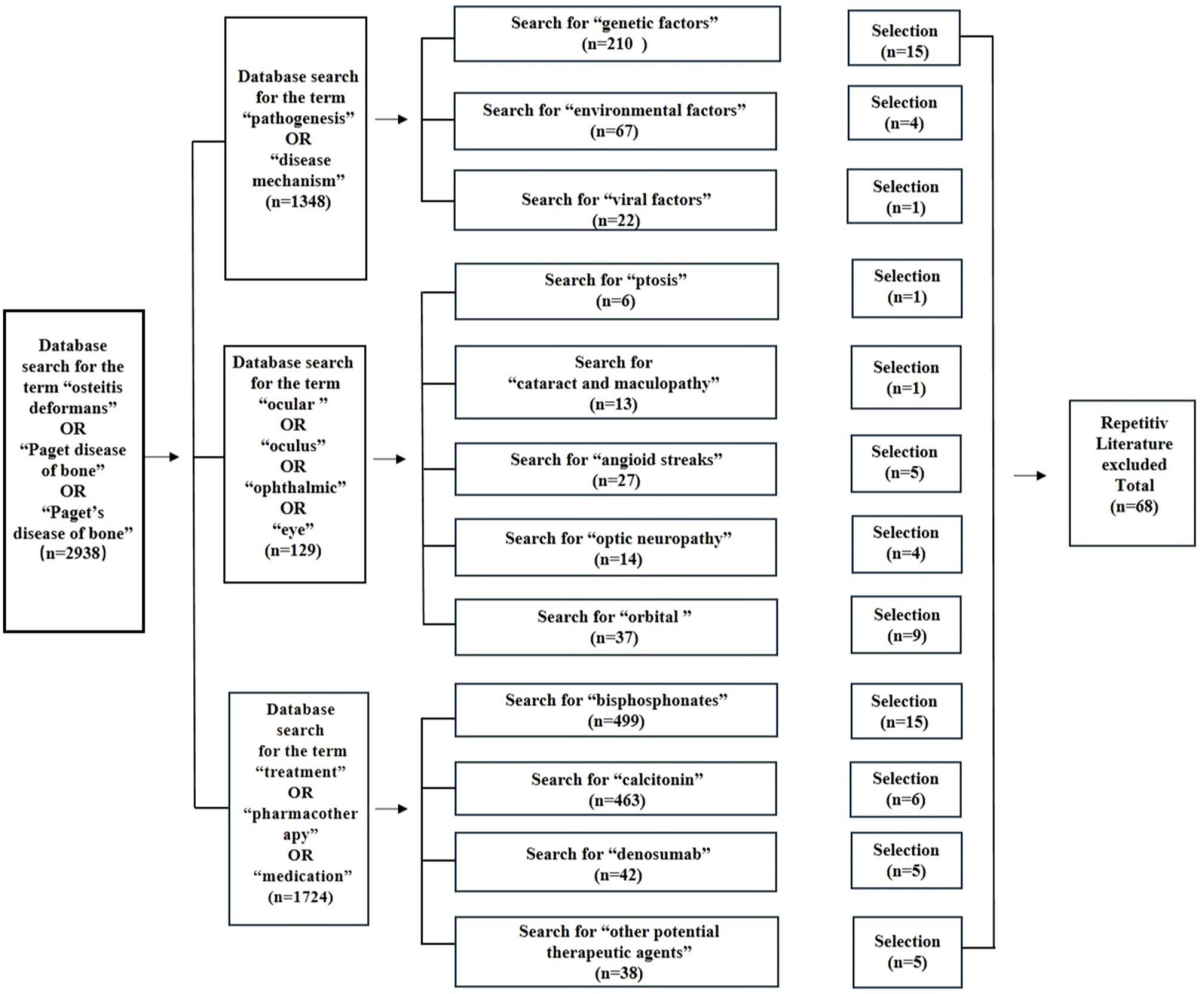
The flow diagram for literature searching

A combined systematic search of PubMed electronic database by using Boolean operators AND and OR was conducted, choosing the following keywords: “Paget’s disease of bone”, “osteitis deformans”, “pathogenesis”, “disease mechanism”, “ocular”, “oculus”, “ophthalmic”, “eye”, “treatment”, “pharmacotherapy”, “medication”, etc. Then all relevant articles in English focusing on Paget’s disease of bone were noticed. After the initial screening of these articles and repetitive literature excluded, only 68 articles were selected. The flow diagram for literature searching is depicted in Fig. 1.

## Introduction

Paget’s disease of bone (PDB), or osteitis deformans, is a chronic focal metabolic bone disorder first described by Sir James Paget in 1876. It is characterized by excessive bone resorption coupled with disordered bone formation [1]. The disease exhibits striking geographical variation in prevalence. In Western populations, PDB is the second most common metabolic bone disease after osteoporosis, with prevalence rates as high as 5.4% reported in the United Kingdom. In contrast, the incidence is remarkably low across Asia, exemplified by rates of only 0.00028% in Japan and 0.38–1.26 per 100,000 individuals in South Korea [2]. Early-stage PDB is frequently asymptomatic. Bone pain is the most common presenting complaint, while other clinical features include bone deformity, pathological fractures, protrusio acetabuli, spinal stenosis, secondary osteoarthritis in adjacent joints, and neurosensory hearing loss. Less frequently, cardiovascular and metabolic complications occur, and rare sarcomatous transformation is documented [3]. As a systemic disorder, the ocular manifestations of PDB constitute a critically important yet often underestimated dimension of its diagnosis and comprehensive management.

## Pathogenesis of PDB

**Fig. 2:**
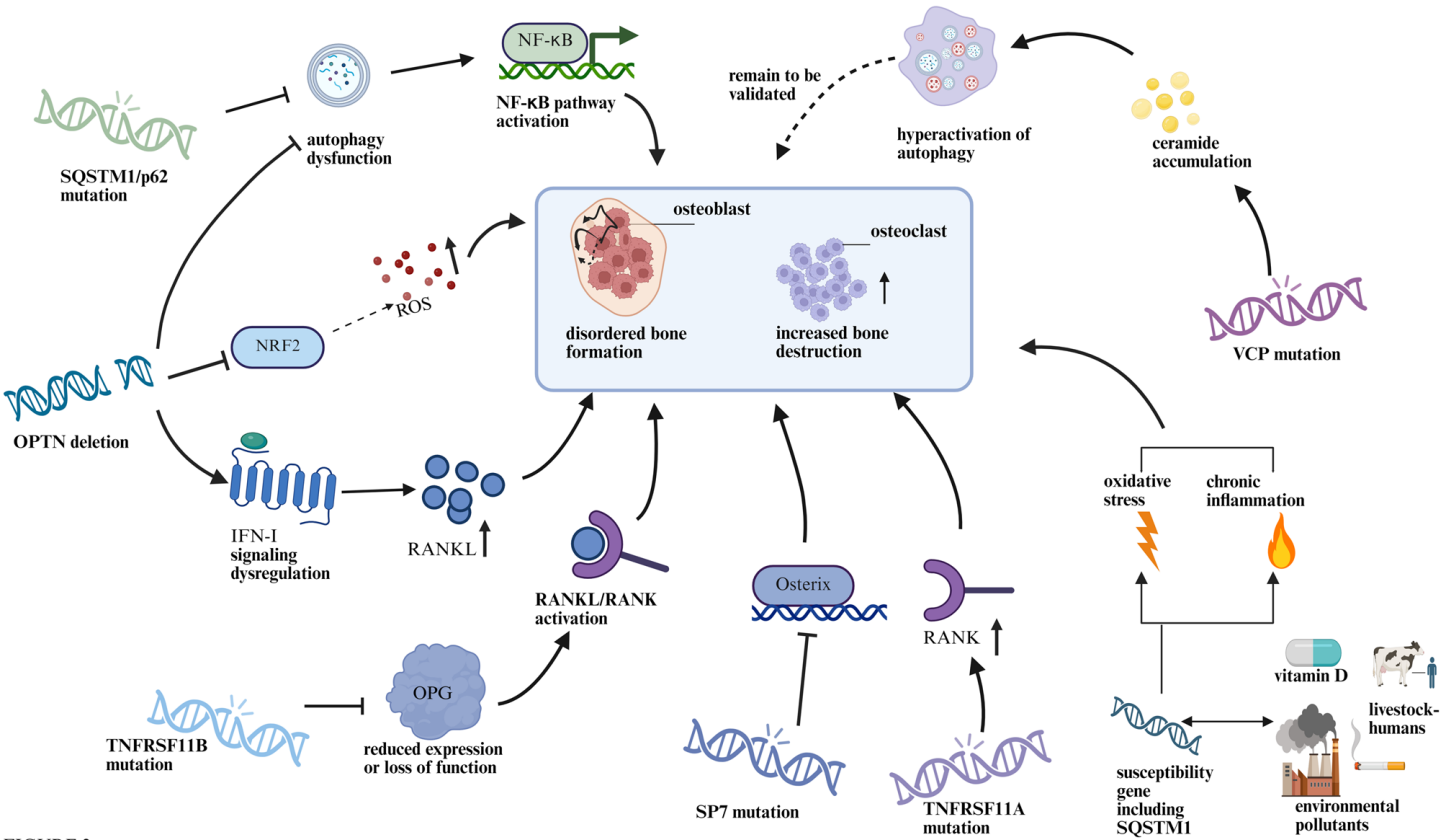
The genetic pathogenesis of PDB

The precise pathogenesis of PDB remains incompletely elucidated. Current research implicates a complex interplay of factors, as summarized in Fig. 2.

### Genetic factors

Genetic predisposition plays a well-established role in PDB. An evaluation of familial clustering in 35 Spanish PDB patients revealed a positive family history in approximately 40%, noting that familial cases typically present earlier, with polyostotic involvement and a male predominance [4]. Another case-control study incorporating sociogenetic indicators (e.g., surnames, consanguinity) suggested that vascular calcification might serve as a biological marker for familial PDB, offering a potential tool for early identification [5]. Consequently, a comprehensive assessment integrating family history and phenotypic traits is clinically valuable for evaluating genetic susceptibility.

#### Sequestosome 1 (*SQSTM1*)

Mutations in the *SQSTM1* gene are a major genetic cause of PDB. These mutations are identified in approximately 25%-40% of familial cases and 10%-15% of sporadic cases [4]. Common variants include missense and truncating mutations. The majority of these pathogenic mutations, predominantly clustered within the ubiquitin-associated (UBA) domain of the encoded p62 protein, impairing its ubiquitin-binding function. This disruption interferes with p62’s role in autophagy and lead to constitutive activation of the NF-κB signaling pathway, thereby enhancing osteoclast activity [6]. Genotype-phenotype analyses indicate that nonsense mutations truncating the UBA domain are associated with more extensive and severe skeletal disease compared to missense mutations. Importantly, *SQSTM1* mutations exhibit incomplete penetrance and significant phenotypic heterogeneity, even among family members carrying the identical variant [4]. 

Thus, *SQSTM1* mutations account for only a proportion of PDB cases, particularly among sporadic patients, implying contributions from other genetic or environmental factors. Recent studies also link *SQSTM1* mutations to multi-system disorders like amyotrophic lateral sclerosis (ALS), indicating that p62 dysfunction has effects beyond the skeleton [7]. While *SQSTM1* testing can aid in identifying at-risk individuals, its predictive utility is limited by incomplete penetrance and phenotypic heterogeneity.

#### Optineurin (*OPTN*)

*OPTN* is a multifunctional protein central to maintaining bone homeostasis. It bidirectionally regulates bone turnover by inhibiting osteoclastogenesis and promoting osteogenic differentiation of bone marrow stromal cells (BMSCs), integrating key pathways including NF-κB, type I interferon (IFN-β), and the antioxidant NRF2 axis [8]. Reduced *OPTN* function is considered an independent risk factor for PDB.

Studies using systemic *OPTN* knockout mice have detailed the mechanism by which *OPTN* deficiency drives PDB-like pathology. *OPTN* loss disrupts type I interferon (IFN-I) signaling, which heightens osteoclast sensitivity to RANKL, promoting differentiation and hyper-resorptive activity, while simultaneously bone formation by inhibiting the key osteogenic transcription factor Osterix. Longitudinal analysis confirmed that *OPTN* deficiency induces age-dependent, progressive bone lesions mirroring human PDB [9]. 

Further research indicates that *OPTN* stabilizing the transcription factor *NRF2* by competitively binding *KEAP1*, thereby activating the cellular antioxidant response. *OPTN* deficiency accelerates *NRF2* ubiquitination and degradation, leading to reactive oxygen species (ROS) accumulation in bone tissue, which exacerbates bone resorption and aging. Notably, bone levels of both *OPTN* and *NRF2* proteins are substantially higher in aged versus young wild-type mice (~ 8-fold and ~ 6-fold, respectively), confirming age-enhanced activation of this pathway and elucidating a mechanism for the age-dependent risk of PDB [10]. 

Additionally, advanced image analysis techniques have shown that *OPTN* mutation disrupts mitophagy, causing mitochondrial network fragmentation and impaired connectivity, revealing a pathogenic mechanism at the level of organelle quality control [11]. 

Collectively, these findings position *OPTN* as a core regulator of bone homeostasis. Its loss contributes to PDB through multiple intertwined mechanisms—disrupting immune signaling, antioxidant defenses, and organellar integrity. This understanding not only explains the age dependency of PDB but, more importantly, shifts the therapeutic paradigm from downstream osteoclast suppression to the potential for precise, early intervention targeting upstream signaling pathways.

#### *TNFRSF11B*, *TNFRSF11A*, and *SP7*

Juvenile Paget’s disease (JPD) and classic PDB share the hallmark of hyperactive osteoclasts leading to increased bone resorption and disorganized formation. However, JPD is typically an autosomal recessive disorder, with higher incidence in consanguineous populations, and presents in childhood with skeletal deformity, pathological fractures, and extra-skeletal features [12]. Its core etiology involves loss-of-function mutations in the *TNFRSF11B* gene encoding osteoprotegerin (OPG). These mutations render OPG incapable of inhibiting the RANKL/RANK pathway, resulting in excessive osteoclast activation that severely disrupts skeletal development and remodeling [13]. 

Not all JPD-like phenotypes are linked to *TNFRSF11B* mutations, highlighting significant genetic heterogeneity. For example, a clinically typical Bolivian girl lacked a *TNFRSF11B* mutation but harbored a heterozygous tandem duplication mutation (87dup15) in exon 1 of the *TNFRSF11A*, predicted to cause a pentapeptide extension in RANK and an expansive skeletal hyperphosphatasia phenotype. Another female infant presented with non-traumatic fractures, cranial deformities, and hearing loss, developing generalized osteosclerosis by age 15; exome sequencing revealed a de novo heterozygous missense mutation in *SP7* (c.926 C > G; p.S309W). Based on such findings, Whyte et al. proposed a molecular classification: JPD-1 (*TNFRSF11B*, autosomal recessive); JPD-2 (*TNFRSF11A* signal peptide duplication, autosomal dominant); JPD-4 (SP7 missense mutation, autosomal dominant); and JPD-3 (unknown genetic basis) [14]. 

This classification expands understanding of RANK/RANKL/OPG-related disorders and reveals a polygenic background for JPD. It requires ongoing refinement as new gene-phenotype associations emerge. The divergent skeletal outcomes from different mutation types in the same gene (e.g., activating vs. inactivating *TNFRSF11A* mutations) underscore that the direction of functional impact critically shapes the phenotype. Most mutations lack thorough functional characterization; how they alter protein function, signaling, and cellular behavior, particularly the pathway from *SP7* mutations to high bone turnover_remains unclear. The autosomal dominant inheritance of JPD-2 and JPD-4, contrasting with recessive JPD-1, indicate that diagnosis must integrate genetic testing and not rely solely on family history.

#### Valosin-containing protein (*VCP*)

Autosomal dominant missense mutations in the *VCP* gene cause *VCP*-associated multisystem proteinopathy (*VCP*-*MSP*), a degenerative condition that can include inclusion body myopathy (IBM), PDB, and frontotemporal dementia (FTD) in various combinations [15]. Recent research focusing on lipid metabolism utilized a homozygous VCP< R155H/R155H> knock-in mouse model and patient iPSC-derived myoblasts to demonstrate that this mutation activates serine palmitoyltransferase (SPT), promoting de novo ceramide synthesis. The resulting ceramide accumulation triggers excessive autophagy and TDP-43 protein mislocalization. SPT inhibitors (e.g., L-cycloserine, myriocin, and ARN14494) effectively reduced levels of autophagy markers p62 and LC3B in these models [16]. 

While these studies offer important mechanistic insights into *VCP*-*MSP*, research on the ceramide pathway has primarily involved muscle models. Validation within the bone microenvironment, specifically in osteoclasts is lacking, and how *VCP* mutations specifically affect bone-remodeling cells remains unclear.

#### Other genes

Beyond *SQSTM1* and *VCP*, other genes contribute to PDB’s polygenic background. Germline mutations in *ZNF687* are associated with PDB co-occurrence with giant cell tumor (GCT) of bone. *ZNF687* encodes a transcription factor involved in DNA damage response, particularly in suppressing gene expression near double-strand breaks and promoting homologous recombination repair [17]. Genome-wide association studies (GWAS) have identified common variants in genes like *CSF1*, *TNFRSF11A*, and *TM7SF4*, which influence osteoclast differentiation/function and may modestly increase PDB risk [18]. A study of sporadic PDB in a Chinese population suggested potential novel susceptibility genes, including *WNT16*, *RYR3*, *RYR1*, *NUP205*, *CAPN2*, and *NUP214*, further broadening the genetic landscape [19]. 

These findings underscore the role of ploygenic interactions in PDB susceptibility, where variants in genes affecting DNA repair, osteoclast biology, and signaling may collectively predispose individuals, with disease triggered by environmental factors. However, many of these associations are preliminary and require independent replication andfunctional validation. Future integrated multi-omics studies are needed to define their precise roles in bone biology.

### Environmental factors

Environmental factors are significant contributors to PDB pathogenesis, both independently and through interaction with genetic susceptibility.

A large study of 2,342 Spanish PDB patients found higher incidence in rural areas, with disease risk moderately with dairy cattle density. The sustained decline in PDB incidence over recent decades also parallels changes in human-livestock interactions, suggesting a non-genetic component [20]. However, this study relied on primary care records susceptible to bias, lacked a matched control group for sporadic cases, and its observational design cannot prove causality.

Vitamin D deficiency is another potential environmental risk, being more prevalent in PDB patients than in healthy populations [21]. This correlation does not establish causation, and confounding factorslike sunlight exposure were not fully assessed. Thus, a causal link requires validation through prospective studies and mechanistic investigations.

An investigation of 140 French-Canadian PDB patients (39 with the SQSTM1 p. Pro392Leu mutation, 113 controls) assessed air pollution and indoor pollutants (heating fuels, tobacco smoke). Cigarette smoke condensate and heavy metals inhibited osteoclast morphology and resorptive activity. Patient osteoclasts showed upregulated gene expression with cadmium, increased *SQSTM1* protein with bismuth/tobacco smoke, and elevated oxidative stress [22]. This suggests environmental pollutantscan modulate osteoclast function and, by interacting with susceptibility genes like *SQSTM1*, induce oxidative stress and autophagy dysregulation. Historically, PDB emerge as prevalent in late-19th century industrialized Britain, its spatiotemporal distribution closely tied to domestic coal use. Incidence declined with reduced coal heating in the 20th century. Migration studies showing high PDB rates among UK migrants to North America/Australia, but rarity in indigenous populations, strongly implicate specific environmental triggers like coal combustion products [23]. 

In summary, environmental factors are key triggers in PDB. Their core mechanism likely involves interaction with a permissive genetic background, disrupting bone remodeling via induced oxidative stress and inflammation. The effect size depends strongly on individual genetics (e.g., SQSTM1 status), explaining why only some exposed individuals develop disease. Future prospective cohorts integrating exposomic and genetic data are needed to quantify risks and delineate molecular pathways, informing primary prevention and early intervention strategies.

### Viral factors

An early etiological hypothesis implicated persistent paramyxoviral infection (e.g., measles virus, respiratory syncytial virus) in osteoclasts as a cause of PDB. However, recent serological evidence strongly refutes this. Visconti et al. found no significant difference in serum antibody levels against multiple paramyxoviruses between PDB patients and healthy controls [24]. It challenges the notion that persistent viral infection is a common cause, suggesting prior associations may have been incidental.

Early studies relied on less specific methods, such as identifyingviral inclusion-like structures in osteoclast nuclei or immunohistochemical detection of viral antigens, which are prone to false positives and cannot distinguish cause from effect. The negative serological data, derived from robust quantitative methods, are highly valuable. Consequently, future etiological research should move beyond the viral model to focus on interactions between other environmental triggers and genetic susceptibility.

In summary, the pathogenesis of PDB arises from the convergent interplay of genetic susceptibility and environmental exposures, with viral etiologies not supported by contemporary evidence. Genetic factors, primarily through germline mutations in genes such as *SQSTM1*, *TNFRSF11B*, *OPTN*, and *VCP*, establish a cell-autonomous dysfunction in bone cells, creating a necessary predisposing state. These mutations also function as primary determinants of disease phenotype, governing severity (e.g., *SQSTM1* truncations), syndromic form (e.g., *TNFRSF11B* in JPD), and specific complication risks (e.g., *ZNF687* with GCT).

Environmental factors are implicated as the principal disease triggers. Epidemiological and historical data associate specific agents (e.g., historical coal combustion products) with disease initiation, acting as a likely “second hit” in genetically primed individuals. Furthermore, in vitro evidence suggests that pollutants (e.g., heavy metals) may interact with susceptibility genotypes (e.g., *SQSTM1*), potentially modulating osteoclast activity through synergistic stress pathways, thereby influencing disease onset or local progression.

Thus, disease initiation appears to require the synergistic contribution of both genetic susceptibility and permissive environmental exposure. Once established, the specific genetic variant is the dominant driver of disease presentation, trajectory, and complication profile. This multi-factorial framework, summarizing the roles of genetic and environmental factors as susceptibility determinants, triggers, and phenotype modifiers, is presented in Table 1.

**Table 1:** Relative roles of etiological factors in the initiation and progression of PDB

Factor Category	Primary Role in Pathogenesis	Mechanism of Contribution	Nature of Role (Trigger / Modifier)
Genetic (*SQSTM1*, *OPTN*)	Susceptibility & Initiation	Germline mutations cause inherent dysfunction in osteoclast/osteoblast signaling pathways (NF-κB, autophagy, IFN), lowering the threshold for abnormal remodeling	Necessary Susceptibility Factor & Primary Phenotype Modifier
Genetic (*TNFRSF11B*, *ZNF687*)	Phenotype Determination	Specific mutations dictate distinct clinical syndromes (JPD) or complication risks (GCT), guiding disease expression	Primary Phenotype Modifier
Environmental (historical coal, livestock)	Disease Trigger	Provide the external “second hit” that initiates focal osteoclast activation in a genetically primed skeletal site	Primary Trigger
Environmental (pollutants, smoking)	Risk & Severity Modifier	May interact with susceptibility genes (*SQSTM1*), exacerbating oxidative stress/dysautophagy to modulate lesion onset or aggressiveness	Interacting Trigger & Progression Modifier
Environmental (rural residence, Vit D deficiency)	Population Risk Factor	Associated with increased odds of disease, likely through broad, non-specific mechanisms or confounding	Weak Correlative Modifier
Viral (Measles, RSV)	Not Supported	No serological evidence of causal association; historical links likely coincidental or artefactual	Not a Trigger or Modifier

Abbreviations: Paget’s disease of bone (PDB), Sequestosome1(SQSTM1), Optineurin (OPTN), Nuclear Factor Kappa B (NF-κB), Interferon (IFN), Tumor Necrosis Factor Receptor Superfamily Member 11B (TNFRSF11B), Zinc Finger Protein 687 (ZNF687), Juvenile Paget’s Disease (JPD), Giant Cell Tumor (GGT), Vitamin D (Vit D), Respiratory Syncytial Virus (RSV)

## Ocular manifestations of PDB

**Fig. 3:**
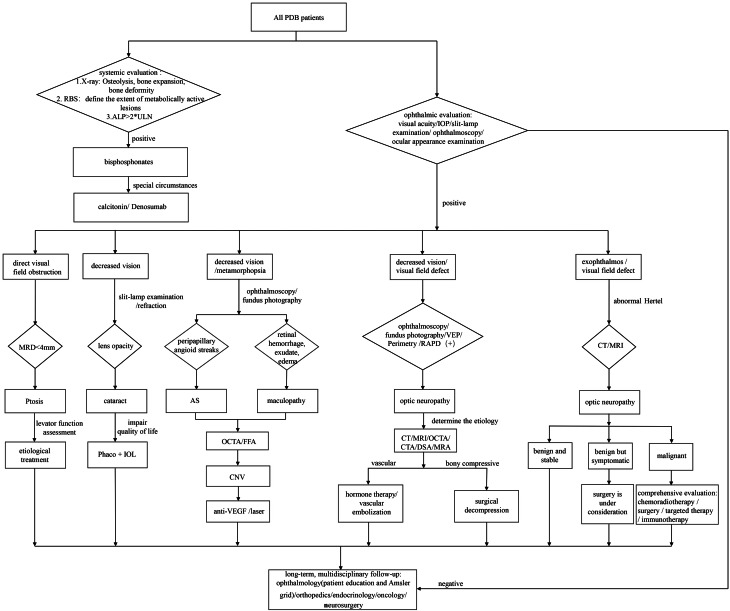
The diagnostic and therapeutic pathway for PDB-related ocular complications. Key abbreviations used: Paget‘s disease of bone(PDB), Radionuclide Bone Scintigraphy(RBS), alkaline phosphatase(ALP), Upper Limit of Normal(ULN), intraocular pressure(IOP), Margin Reflex Distance (MRD), visual evoked potential(VEP), Relative Afferent Pupillary Defect (RAPD), computed tomography(CT), magnetic resonance imaging(MRI), angioid streaks(AS), Phacoemulsification(Phaco), intraocular lens (IOL), optical coherence tomography angiography(OCTA), fluorescein fundus angiography(FFA), choroidal neovascularization(CNV), computed tomography angiography(CTA), digital subtraction angiography(DSA), magnetic resonance angiography(MRA), anti-vascular endothelial growth factor(anti-VEGF)

The diverse ocular manifestations of PDB necessitate a management approach that transcends specialty boundaries, relying on a multidisciplinary team (MDT) encompassing orthopedics, oncology, and ophthalmology. Figure 3 outlines this integrated diagnostic and therapeutic pathway. This flowchart delineates a stepwise diagnostic pathway to differentiate the causes of visual complaints and clarifies the decision-making criteria for initiating systemic anti-resorptive therapy versus proceeding with ocular-specific interventions.

### Ptosis

Orbital bone involvement in PDB can cause ptosis. A specific mechanism is the formation of a PDB-related giant cell tumor (GCT) within the orbit, which directly compresses the levator palpebrae superioris muscle [25]. Thus, abnormal orbital bone remodeling or secondary neoplastic lesions constitute the direct pathological basis. The active pathological bone metabolism and associated chronic inflammatory microenvironment may further impair muscle structure and function through mass effect, tissue infiltration, or neurovascular compromise. Treatment focuses on addressing the underlying cause.

### Cataract and maculopathy

A study by Dabbs et al. evaluating ocular complications in 70 PDB patients reported cataract and macular degeneration incidences of 39% and 24.3%, respectively [26]. However, this study did not establish a direct causal link between these intraocular pathologies and PDB.

### Angioid streaks (AS)

AS are linear cracks in a calcified, brittle Bruch’s membrane, representing a rare retinal finding and a recognized ocular complication of PDB [27]. The principal visual threat is rupture or secondary choroidal neovascularization (CNV), leading to irreversible visual loss.

Historically, 8%-15% of PDB patients were reported to develop AS [26]. Conversely, among all patients presenting with AS, only about 0.3% have coexisting PDB [28]. This indicates that while PDB is an important etiology for AS, AS is more commonly associated with other systemic diseases like s pseudoxanthoma elasticum. It underscores the need for routine ocular screening in PDB patients and the importance of considering PDB in the differential diagnosis ofAS, especially in older patients or those with skeletal symptoms.

Notably, JPD also carries a retinopathy risk, characterized by progressive AS, potentially linked to Bruch’s membrane calcification from dysregulated osteoprotegerin signaling [29]. This suggests PDB and its ocular complications may share a common pathophysiological basis of “disordered connective tissue/extracellular matrix metabolism”.

Management of asymptomatic AS primarily involves observation. Upon CNV development, prompt intravitreal anti-vascular endothelial growth factor (VEGF) therapy is indicated. Combination therapy with photodynamic therapy and anti-VEGF agents (e.g., bevacizumab) can effectively suppress neovascular activity. Patients are also advised to wear protective eyewear and avoid ocular trauma to minimize Bruch’s membrane rupture risk [30]. 

Given these risks, a systematic, risk-stratified protocol encompassing screening, prevention, and intervention is warranted. For all PDB patients, the focus is “Screening and Prevention” via annual dilated fundus exams If asymptomatic AS is detected, management shifts to “Monitoring and Alert”, involving patient education and Amsler grid self-monitoring. New visual decline should prompt immediate optical coherence tomography (OCT) and fluorescein angiography (FFA) to assess for CNV activity. Confirmed active CNV warrants immediate “Intervention and Rescue” with anti-VEGF therapy.

### Optic neuropathy

The mechanisms of g optic neuropathy in PDB are multifaceted, extending beyond simple bony compression.

Bony compression is one established mechanism, particularly in JPD where cranial overgrowth can compress the orbital apex optic nerve, causing pallor and atrophy [29]. Studies also find visual field defects in approximately 40.9% of PDB patients, some with radiologic evidence of optic canal stenosis [31]. 

However, vascular mechanisms appear central. A pivotal case report described an adult PDB patient with severe progressive vision loss without canal stenosis, whose vision improved markedly after pamidronate and dexamethasone [32]. This response strongly implicates optic nerve ischemia from a vascular “steal” phenomenon rather than mechanical compression. Supporting this, only a minority of patients with field defects have definitive canal stenosis [31]. Another illustrative case involved a 73-year-old man with PDB-related orbital disease, decreased vision, proptosis, and periorbital swelling. Imaging showed stable bony lesions, but angiography confirmed hypervascularity. Ocular examination revealed “corkscrew” conjunctival vessels, serous choroidal/retinal detachment, optic disc hyperemia, and tortuous veins. Reducing this abnormal flow via vascular embolization resolved the subretinal fluid, demonstrating that a high-flow state alone can cause orbital congestion and visual impairment [33]. 

Thus, PDB-related optic neuropathy constitutes a spectrum involving vascular and compressive factors, with mechanisms like bony steal and vascular congestion likely predominant. Compression is more typical in severe craniofacial JPD or select adult cases. Direct invasion by pagetic tissue must also be considered. Critically, the mechanism dictates treatment: response to anti-resorptive/corticosteroids supports a hypermetabolic/vascular inflammatory basis, while embolization targets hyperperfusion, and surgical decompression is reserved for true mechanical compression. Therefore, evaluation must extend beyond structural imaging (CT/MRI) to include assessments of blood flow and function (e.g., angiography, OCT), guiding mechanism-based treatment.

### Orbital disease

Orbital involvement in PDB leads to diverse structural and space-occupying lesions.Proptosis is the hallmark manifestation, primarily via: (1) Bony Compression: Craniofacial hyperostosis reduces orbital volume, mechanically displacing the globe forward (e.g., a case with 22 mm proptosis) [34]. (2) Hemodynamic Factors: The hypervascular pagetic lesions can cause orbital tissue congestion and volume increase(e.g., progressive proptosis with stable bone lesions but abnormal vasculature) [33]. 

PDB can also cause enophthalmos, theorized to result from anterior displacement of the superior orbital rim due to anterior cranial fossa thickening, creating a relative posterior globes position [35]. 

Localized orbital mass is another presentation, with cases reported of painless supraorbital swelling and proptosis confirmed as PDB by histopathology, indicating it can manifest as an isolated orbital mass [36]. 

Rapidly progressive proptosis (weeks to months) should raise high suspicion of secondary malignant (e.g., osteosarcoma, chondrosarcoma) or, less often, benign tumors like GCT. A Japanese national study a bimodal age distribution for PDB-associated osteosarcoma (peaks: 10–20 and 70–80 years). Elderly patients often have axial (including craniofacial) tumors diagnosed at advanced stages with poor prognosis [37]. These malignancies, favoring elderly women, are frequently resistant to radio-chemotherapy, leading to death within months [38, 39]. Rarely, such malignancies occur in infancy, with report cases showing progressive drug resistance and inoperability despite aggressive therapy [40]. In contrast, benign GCTs generally have a good prognosis after resection, possibly arising from systemic stimulation of osteoclast precursors by PDB [25]. 

Therefore, orbital Therefore PDB spans a spectrum from benign structural change to lethal malignancy. Clinical management hinges on accurately identifying the dominant process: slow bone remodeling, active hemodynamic disturbance, or rapid malignant transformation. Future efforts should aim to develop better screening tools for early detection of malignancy and explore novel therapies for PDB-associated cancers.

The ocular complications in PDB arise not in isolation but through specific pathophysiological pathways originating from the systemic dysregulation of bone remodeling. These mechanisms can be categorized into direct mechanical effects, secondary vascular phenomena, and systemic metabolic dysregulation extending to ocular tissues. The predominant mechanism varies by the type of ocular involvement, which dictates appropriate clinical management. Table 2 summarizes the proposed molecular, cellular, and hemodynamic pathways linking pagetic bone disease to its major ocular manifestations.

**Table 2:** Proposed pathophysiological mechanisms linking PDB to ocular complications

Ocular Complication	Dominant Mechanism	Pathophysiological Process	Key Molecular/Cellular Pathways
Orbital Deformity (Proptosis, Enophthalmos)	Bone Remodeling/ Mechanical	Disordered osteoclastic-osteoblastic activity leads to expansion and deformation of orbital bones, altering orbital volume and anatomy	Dysregulation of osteoclast-osteoblast coupling (RANKL/OPG, SQSTM1/p62, OPTN signal paths)
Ptosis secondary to Orbital GCT	Bone Remodeling/ Mechanical (Mass Effect)	PDB-associated proliferation of osteoclast precursors forms a benign, space-occupying tumor that mechanically compresses the levator palpebrae superioris muscle	Local clonal expansion of osteoclast precursors driven by the systemic PDB milieu; potential role of *ZNF687* mutations
Compressive Optic Neuropathy (JPD)	Bone Remodeling/ Mechanical	Craniofacial hyperostosis, particularly of the sphenoid bone, results in stenosis of the bony optic canal, causing direct compression and ischemia of the optic nerve	Uncontrolled osteoblastic bone formation at specific cranial sites
Vascular Optic Neuropathy	Vascular/ Hemodynamic	Hypervascularity of pagetic bone creates a low-resistance shunt (“steal” phenomenon), diverting blood flow from the microvasculature of the optic nerve head and leading to ischemia	VEGF-mediated pathological angiogenesis within pagetic bone; altered local hemodynamics
Orbital & Conjunctival Congestion	Vascular/ Hemodynamic	Increased blood flow and vascular permeability from adjacent active pagetic bone cause venous engorgement and edema in orbital soft tissues and conjunctiva	Inflammatory cytokine release (e.g., IL-6) and angiogenic drive from the bone microenvironment
AS	Metabolic/ Systemic	Systemic dysregulation of mineral homeostasis, due to loss of calcification inhibitors, results in pathological calcification and increased fragility of Bruch’s membrane	Loss-of-function mutations in *TNFRSF11B* (encoding OPG), a key regulator of extracellular matrix mineralization
Cataract & Maculopathy (Potential Association)	Other/Systemic (Speculative)	A shared systemic milieu of chronic inflammation and oxidative stress may potentially accelerate age-related degenerative processes in the lens and retina	Persistent activation of NF-κB and impairment of the NRF2 antioxidant pathway, as observed in pagetic bone biology

Abbreviations: Paget’s disease of bone (PDB), Receptor Activator of Nuclear Factor Kappa-B Ligand (RANKL), Osteoprotegerin (OPG), Sequestosome1 (SQSTM1), Optineurin (OPTN), Giant Cell Tumor (GGT), Zinc Finger Protein 687 (ZNF687), Juvenile Paget’s Disease (JPD), Vascular Endothelial Growth Factor (VEGF), Interleukin-6 (IL-6), Angioid Streak s(AS), Tumor Necrosis Factor Receptor Superfamily Member 11B (TNFRSF11B), Nuclear Factor Kappa B (NF-κB), Nuclear Factor Erythroid 2–Related Factor 2 (NRF2)

## Pharmacological therapy for PDB

Pharmacological intervention is the cornerstone of managing PDB, a systemic disorder.

### Bisphosphonates

Bisphosphonates are the first-line therapy, potently inhibiting osteoclast activity. A retrospective cohort study of 101 PDB patients confirmed the efficacy and sustained biochemical response of zoledronic acid [41]. This class is also effective in JPD; pamidronate controls bone pain, suppresses turnover, improves motor function in children, and has a favorable side-effect profile [42]. Long-term data show a single zoledronic acid infusion can maintain remission for up to 10 years in 86% of treatment-naïve elderly patients, treatment-naïve patients, demonstrating durable control [43]. Given this safety and efficacy profile, the Italian Society for Osteoporosis, Mineral Metabolism, and Skeletal Diseases (SIOMMMS) recommends treatment at diagnosis for most, if not all, cases [44]. 

Notably, bisphosphonates show promise in prevention. Prophylactic zoledronic acid in *SQSTM1* mutations carriers significantly reduced new lesion risk safely, marking a shift from “symptom-driven” to “risk-predictive” management with potential to alter the disease course. Defining the optimal candidate and timing for preventive therapy remains a future challenge [45]. 

Ocular inflammatory adverse events require attention. Zoledronic acid, pamidronate, and others can induce acute anterior uveitis, scleritis, and orbital inflammation, presenting with eye pain, redness, and vision loss [46–49], possibly mediated by γ/δ T-cell dysregulation [50]. 

Beyond ocular events, an acute-phase reaction (self-limiting anemia, thrombocytopenia, lymphopenia) commonly follows zoledronic acid infusion [51]. Risedronate most often causes dyspepsia; rarer effects include facial edema, Stevens-Johnson syndrome, tongue edema, palpitations, and episcleritis [52]. Intravenous bisphosphonates also carry a risk of medication-related osteonecrosis of the jaw (MRONJ), associated with potent antiresorptive therapy [53]. Thus, biomarker-based prediction of adverse reaction risk is an important consideration.

Preclinical insights suggest non-nitrogen-containing bisphosphonates (e.g., etidronate, clodronate), though less potent, may mitigate the acute inflammation induced by nitrogen-containing agents via competitive inhibition of SLC20/SLC34 phosphate transporters, offering a rationale for exploring combination strategies to balance efficacy and safety [54]. 

### Calcitonin

Human calcitonin (hCT), a 32-amino-acid hormone from thyroid parafollicular cells, has long been used to inhibit osteoclast-mediated bone resorption [55]. It also suppresses osteoblast activity and bone turnover [56]. However, hCT is prone to amyloid aggregation in solution (reducing bioactivity), and long-term use may be linked to increased thyroid cancer risk. Targeted modification to reduce amyloidogenicity while preserving function is a promising strategy [57]. More stable, less immunogenic alternatives like salmon (sCT) and eel calcitonin are potential clinical substitutes. Despite advances in delivery (injection, nasal spray, novel oral systems like pectin-chitosan hydrogels), clinical efficacy remains limited [58]. Observational data indicate poor outcomes for patients on calcitonin monotherapy, most eventually requiring switch to more potent drugs like bisphosphonates [59]. Sequential strategies exist, e.g., initial calcitonin providing some symptom relief followed by definitive zoledronic acid therapy [60]. 

With the advent of potent bisphosphonates, calcitonin’s role has diminished. Its current value lies more in specific clinical scenarios: as bridging therapy when IV bisphosphonates are contraindicated (e.g., active infection, renal impairment), or for additional central analgesia for severe bone pain before bisphosphonates take full effect. If successfully developed, novel oral formulations might offer an alternative for the bisphosphonate-intolerant.

### Denosumab

Denosumab, a fully human monoclonal antibody targeting RANKL, inhibits bone resorption and is used for PDB [61]. Case reports support its utility: an elderly woman with cranial PDB achieved headache relief and normalization calcium/ALP after 5 months; [62] two adult JPD patients with *TNFRSF11B* “Balkan” mutation and inadequate bisphosphonate response achieved sustained ALP normalization on long-term denosumab [63]. 

Denosumab also shows potential for PDB-related complications. A patient with PDB, extraosseous GCT, and calcitriol-mediated hypercalcemia experienced controlled hypercalcemia and significant tumor regression on denosumab [64]. 

Thus, denosumab is a potent option for bisphosphonate-resistant or -intolerant patients, with particular advantage in RANKL-driven complications like GCT. However, its reversible mechanism necessitates careful long-term management to mitigate rebound risk upon discontinuation [65]. Future research should optimize sequencing strategies with bisphosphonates.

### Other potential therapeutic agents

Preclinical studies highlight several novel intervention avenues targeting diverse mechanisms.

Based on the *OPTN*-*NRF2*, axis in bone homeostasis, antioxidant therapy shows promise. *OPTN* deficiency-induced bone loss can be alleviated by *NRF2* activators like curcumin. However, in aged Optn−/− mice, curcumin further suppressed osteoblast activity, suggesting a complex interplay in aging where antioxidant treatment might alleviate resorption but also affect compromised formation [10]. Restoring the *NRF2* response may mitigate age-related bone loss, but combination strategies may be needed.

Since *OPTN* negatively regulates type I interferon signaling to restrain osteoclasts, targeted inhibition of this pathway could restore homeostasis, though requires rigorous safety evaluation to avoid immunodeficiency from systemic inhibition [9]. 

Broadly, gut microbiota-derived short-chain fatty acids can regulate bone remodeling, exerting both anti-resorptive and anabolic effects [66]. Clinically, specific probiotic supplementation (e.g., Lactobacillus reuteri 6475) slowed bone loss in postmenopausal women, introducing the microbiome as a novel systemic modulator of bone metabolism [67, 68]. 

In summary, PDB drug discovery is evolving from traditional anti-resorptive towards precision, system-based strategies targeting multiple pathways. Future paradigms may involve combinations (e.g., probiotics for baseline homeostasis plus targeted agents for specific defects) to correct the abnormal remodeling program at its root. Systemic treatments can directly reverse specific, metabolism-driven ocular symptoms but primarily serve to control the systemic disease and prevent progression of structural damage. Ocular-specific therapies are necessary adjuncts to address localized, irreversible structural issues or distinct ocular vascular pathologies. Their interaction is best characterized as sequential and complementary, aiming for comprehensive disease control. (Table 3)

**Table 3:** Therapeutic strategy for ocular complications in PDB based on pathogenic mechanism

Ocular Complication	Dominant Mechanism	Role of Systemic Therapy (Bis/Denosumab)	Necessity of Ocular-Specific Therapy	Therapeutic Synergy & Timing
Vascular Optic Neuropathy	Vascular “Steal” / Hyperemia	Direct & Primary: Suppresses pagetic hypermetabolism/hypervascularity to reverse ischemia	Adjunct: Corticosteroids may be used acutely to reduce optic nerve edema/inflammation; severe cases may consider vascular embolization	Concurrent/Sequential: Start systemic anti-resorptive urgently; consider short-term steroids for acute presentation
Compressive Optic Neuropathy	Bony Stenosis / Mechanical	Foundational/Preventive: Halts progression of hyperostosis; does not reverse existing compression	Necessary & Definitive: Surgical decompression is indicated for progressive or severe compression	Sequential: Optimize systemic control preoperatively to reduce bleeding risk; surgery addresses fixed anatomy
Orbital GCT causing Ptosis	Mass Effect	Variable/Adjuvant: Denosumab may induce tumor regression; bisphosphonates control background PDB activity	Necessary & Definitive: Surgical excision is primary treatment for symptomatic mass effect	Combined/Neoadjuvant: Consider denosumab to shrink tumor preoperatively; surgery for definitive removal
AS with CNV	Local Neovascularization	Indirect/Supportive: Manages systemic disease but has no direct effect on CNV	Necessary & Primary: Intravitreal anti-VEGF injections are standard care for active CNV	Parallel: Treat systemic disease and ocular CNV independently but concurrently
Cataract	Age-related / Multifactorial	No Role	Necessary & Definitive: Cataract surgery when visually significant	Independent: Ocular surgery proceeds based on ophthalmic indication, independent of PDB status if bone is inactive

*Abbreviations: Paget’s disease of bone (PDB)*, Bisphosphonates (Bps), *Giant Cell Tumor (GGT)*,* Angioid Streaks (AS)*,* Choroidal Neovascularization (CNV)*,* Anti-Vascular Endothelial Growth Factor (*anti-*VEGF)*

## Evaluation of evidence: causality versus correlation in PDB pathogenesis

To provide a critical appraisal of the associations discussed in this review and to explicitly address the distinction between correlation and causation, we have applied an evidence hierarchy framework centered on the strength of causal inference. This framework classifies the evidence linking genetic, environmental, and viral factors to both systemic PDB and its ocular complications into four distinct levels, as outlined in Table 4.

**Table 4:** Evidence hierarchy and critical appraisal of key pathogenetic and clinico-pathological associations in PDB

Association	Evidence Level	Study Designs Supporting Association	Supporting Rationale	Major Confounders / Limitations	Key References
**Genetic factors**					
Severe, polyostotic PDB in *SQSTM1* mutation carriers	Level 2: Strong Mechanistic	Family-based linkage studies; case-control genetic association studies; in vitro functional assays (osteoclast cultures, autophagy flux); genetically engineered mouse models (*SQSTM1* knock-in/knockout)	Well-defined molecular cascade (NF-κB/autophagy dysregulation) leading to the aggressive bone phenotype; orbital involvement is a direct secondary consequence	Incomplete penetrance & significant phenotypic heterogeneity within families limit clinical predictive value. Majority of mechanistic data are derived from non-human models or cellular systems; direct validation in human bone in vivo remains partial. Potential ascertainment bias in studies focused on severe familial cases	*Gennari* et al.,* 2022; Shaik* et al.,* 2021*
AS in *TNFRSF11B*-related JPD	Level 2: Strong Mechanistic	Case series of genetically confirmed JPD patients with detailed ophthalmological phenotyping; genetic sequencing studies; mechanistic extrapolation from OPG’s known physiological role	OPG’s known role in inhibiting vascular calcification provides a direct, plausible pathway from genetic defect to Bruch’s membrane pathology	Evidence is based almost exclusively on the rare, monogenic JPD population; its direct applicability to the pathogenesis of AS in classic, late-onset PDB is less certain. Small sample sizes inherent to ultra-rare disorders	*Kerr* et al.,* 2010*
**Environmental factors**					
Rural residence/cattle density and PDB risk	Level 3/4: Primarily Correlative	Ecological (population-level) studies; retrospective analyses of large primary care or administrative health databases	Ecological association with no specified molecular mechanism; highly susceptible to confounding	High risk of confounding: Association may be attributable to unmeasured factors correlated with rural residence (specific occupational exposures, diet, socioeconomic status). Ecological fallacy. Dependent on accuracy and consistency of diagnostic coding in electronic records	*Rebollo-Najera* et al.,* 2025*
Vit D deficiency and PDB.	Level 4: Purely Correlative	Cross-sectional observational studies comparing serum 25-hydroxyvitamin D levels between PDB patients and healthy or disease controls	Observational association only; high likelihood of reverse causality (reduced mobility in PDB) or confounding	High probability of reverse causality: PDB-related skeletal pain and disability likely reduce outdoor activity and sun exposure. Confounding by age, season, latitude, body mass index, and chronic kidney disease. No prospective or interventional data to support a causal role	*Rendina* et al.,* 2019*
*SQSTM1* mutation and pollutant (Cd, smoke) interaction	Level 3: Correlative with Plausible Mechanism	In vitro experiments using osteoclasts differentiated from PBMCs of *SQSTM1* mutation carriers and controls, exposed to heavy metals or cigarette smoke condensate	In vitro data shows altered osteoclast biology; proposed oxidative stress mechanism is plausible but lacks direct in vivo causal proof in humans	Evidence is preclinical (in vitro only). Doses and exposure durations may not reflect real-world human environmental exposure Small patient-derived cell sample sizes. Lacks in vivo validation in animal or human studies to confirm this interaction causes clinical disease	*Numan* et al.,* 2019*
Historical coal exposure and PDB emergence	Level 3: Correlative with Plausible Mechanism	Historical ecological analyses examining spatial-temporal disease patterns alongside fuel use; migration studies comparing disease prevalence in migrants from high-risk areas to native populations	Strong spatiotemporal correlation; coal-derived toxins (heavy metals, fluorides) are biologically plausible triggers, but direct evidence is historical/epidemiological	Evidence is circumstantial and ecological; cannot establish causality at the individual level. Relies on the accuracy of historical disease and exposure data. The precise pathogenic constituent(s) of coal smoke remain unidentified	*Cundy*, *2024*
**Direct structural/compressive ocular lesions**					
Ptosis due to PDB-related orbital GCT	Level 1: Causal	Case reports and small surgical series with direct clinico-radiological-pathological correlation (imaging confirming mass, histopathology confirming GCT, surgery relieving symptoms)	Direct mass effect confirmed clinically and radiologically; resection relieves symptoms	Evidence limited to level IV/V case reports/series, subject to publication bias (only remarkable or surgically managed cases are reported). No population-based data on the incidence of this rare complication	*Pecorella* et al.,* 2000*
Compressive optic neuropathy from pagetic OCS	Level 1: Causal	Case series and individual case reports with direct correlation between high-resolution CT/MRI evidence of bony canal narrowing and corresponding clinical/functional optic nerve deficits	Direct imaging evidence of bony compression at the exact site of neurological deficit	Evidence primarily from severe, often syndromic (JPD) cases; may not represent the typical course in adult PDB. Small sample sizes and lack of prospective longitudinal data on progression	*Kerr* et al.,* 2010; Eretto* et al.,* 1984*
**Vascular/metabolic ocular lesions**					
Vascular optic neuropathy from pagetic hypervascularity (“steal” phenomenon)	Level 1/2: Causal / Strong Mechanistic	Level 1: Case reports demonstrating visual improvement following intervention (BP infusion, corticosteroids, or vascular embolization) Level 2: Case reports with angiographic (e.g., DSA, MRA) confirmation of aberrant, high-flow vasculature adjacent to the ischemic nerve	Level 1: Intervention (BPs, embolization) reverses ischemia/edema. Level 2: Angiographic confirmation of abnormal, high-flow vasculature adjacent to the nerve	Evidence is entirely from level IV/V case reports. The diagnosis of “vascular steal” is often inferential, based on a characteristic treatment response rather than direct, quantitative hemodynamic measurement. No controlled studies exist	*Isasi* et al.,* 2002; Yuan* et al.,* 2010*

Abbreviations: Paget’s disease of bone(PDB), Sequestosome1 (SQSTM1), Nuclear Factor Kappa B (NF-κB), Angioid Streaks (AS), Tumor Necrosis Factor Receptor Superfamily Member 11B (TNFRSF11B), Juvenile Paget’s Disease (JPD), Osteoprotegerin (OPG), Vitamin D (Vit D), Cadmium (CD), Peripheral Blood Mononuclear Cells (PBMCs), Giant Cell Tumor (GGT), optic canal stenosis (OCS), Computed Tomography (CT), Magnetic Resonance Imaging (MRI), Bisphosphonate (BP), bisphosphonates (BPs), Digital Subtraction Angiography (DSA), Magnetic Resonance Angiography (MRA)

- Level 1: Direct Causal Evidence. This is established by a clear anatomical-mechanical link (e.g., tumor-induced nerve compression) or a consistent therapeutic response wherein intervening on the presumed cause (e.g., inhibiting bone turnover) directly reverses the clinical effect (e.g., restores vision)
- Level 2: Strong Mechanistic Evidence Supporting Causality. This level encompasses evidence in which a well-characterized biological pathway connects an etiological factor to a pathological outcome, strongly implying a causal relationship. It includes robust data from genetic animal models (e.g., *OPTN* knockout mice) or detailed molecular studies that delineate the intervening pathological steps.
- Level 3: Correlative with Plausible Mechanism. This category includes epidemiological or case-control associations supported by in vitro data or indirect biological plausibility. Such evidence suggests a link meriting further investigation but does not confirm causation, as potential confounding factors or reverse causality cannot be ruled out.
- Level 4: Purely Correlative. This level refers to observational associations for which no established or strongly plausible intermediary biological mechanism has been demonstrated. These relationships may indicate shared underlying risk factors rather than a direct cause-and-effect relationship.

Applying this framework reveals that the most definitive causal links (Level 1) typically involve direct anatomical consequences of pagetic bone remodeling, such as compression of ocular structures. The pathogenic role of specific genetic mutations (e.g., *TNFRSF11B* in angioid streak formation) is supported by strong mechanistic evidence (Level 2). Conversely, most proposed gene-environment interactions and broader epidemiological risk factors (e.g., vitamin D deficiency, rural residence) are currently supported by evidence at the correlative levels (Levels 3–4). This distinction underscores these areas as critical targets for future hypothesis-driven research aimed at establishing causative pathways.

## Limitations of the review

This systematic review synthesizes current evidence on PDB pathogenesis, ocular involvement, and treatment but has inherent limitations. First, much evidence derives from case reports, small series, and retrospective studies, lacking large prospective cohorts or RCTs, necessitating caution in inferring causality and efficacy Second, relevance evidence (level 3–4) far outweighs causal evidence (level 1–2), especially regarding direct links between genetic/environmental factors and specific ocular manifestations. Third, discussed treatment strategies often rely on individual case successes; their long-term efficacy, safety, and optimal regimens require broader clinical validation. Fourth, a significant gap persists in the understanding of the direct molecular and cellular mechanisms that connect systemic pagetic bone remodeling to specific ocular complications. While associations and plausible pathways are described (e.g., OPG dysfunction and Bruch’s membrane calcification for angioid streaks), the evidence remains largely indirect. Crucially, there is a paucity of studies directly analyzing affected human ocular tissues (e.g., histopathology of pagetic orbital bone with adjacent compressed nerve, molecular analysis of Bruch’s membrane in PDB) to validate proposed mechanisms. Furthermore, the relative contribution and potential synergy between different mechanisms (e.g., vascular steal versus direct compression in optic neuropathy) in individual patients are poorly defined, complicating personalized management. Finally, potential publication bias favoring positive results may skew the understanding of rare complications or overestimate treatment effectiveness.

## Conclusion

This review delineates the connections between PDB pathogenesis, its diverse ocular manifestations, and therapeutic strategies. Evidence confirms that abnormal bone remodeling, driven by genetic susceptibility and environmental triggers, is the common pathology underlying both skeletal and specific ocular disease. Therefore, effective PDB management must transcend specialty silos, relying on multidisciplinary collaboration (MDT) among orthopedics, endocrinology, and ophthalmology. Integrating ocular assessment into the standard PDB care pathway and implementing mechanism-based individualized treatment are essential to control skeletal progression while preventingvisual impairment, ultimately enhancing patient quality of life.

To achieve this, future research must prioritize studies designed to establish definitive causal pathways. Key priorities include: (1) Longitudinal, prospective cohort studies of PDB patients with standardized ocular screening, to determine the true incidence and natural history of complications and identify risk factors; (2) Mechanistic studies employing advanced imaging (e.g., high-resolution angiography coupled with OCT), biofluid analysis, and, where possible, targeted histopathology of surgical specimens to bridge systemic biomarkers with local ocular pathology; and (3) Well-designed clinical trials to evaluate whether early, aggressive systemic therapy or novel pathway-targeted agents can prevent the development or progression of sight-threatening ocular complications, moving beyond retrospective case reports.

## Data Availability

Not applicable.
